# Natural resources used as folk cosmeceuticals among rural communities in Vhembe district municipality, Limpopo province, South Africa

**DOI:** 10.1186/s12906-020-2869-x

**Published:** 2020-03-12

**Authors:** Mamokete Venolia Setshego, Adeyemi Oladapo Aremu, Obakeng Mooki, Wilfred Otang-Mbeng

**Affiliations:** 1 Indigenous Knowledge Systems (IKS) Centre, Faculty of Natural and Agricultural Sciences, North-West University, Private Bag X2046, Mmabatho, 2735 South Africa; 2 Food Security and Safety Niche Area, Faculty of Natural and Agricultural Sciences, North-West University, Private Bag X2046, Mmabatho, North West Province South Africa; 3 North West Dingaka Association, Central Mafikeng, 2745 South Africa; 4 School of Biology and Environmental Sciences, Faculty of Agriculture and Natural Sciences, University of Mpumalanga, Private Bag X11283, Mbombela, Mpumalanga Province 1200 South Africa

**Keywords:** Biodiversity, Ethnopharmacognostic, Medicinal plants, Skin diseases, Indigenous knowledge systems

## Abstract

**Background:**

Skin-related diseases affect every individual irrespective of age, gender or social status. Since time immemorial, humans have explored natural resources from their environment for the maintenance of the skin. This explorative survey was conducted to document the natural resources (plant and non-plant materials) used for folk cosmeceuticals by rural communities in Vhembe district municipality, Limpopo province, South Africa.

**Methods:**

The research was conducted in six communities namely: Tshakuma, Shigalo, Tshamutilikwa, Luvhimbi (Masikhwa), Khakhanwa, and Folovhodwe in Vhembe district. Random and convenient sampling was used to access the target population. Semi-structured questionnaires were used to interview 71 participants that comprised traditional practitioners, herbalists and laypeople from the study area. Collected data were analysed using both quantitative (for e.g. frequency, use-value and relative frequency of citation) and qualitative (thematic) analytical methods.

**Results:**

A total of 52 plants from 27 families and 22 non-plant materials were used as folk cosmeceuticals in the study area. The most cited plants included *Dicerocaryum zanguebarium* (Pedaliaceae), *Ricinus communis* (Euphorbiaceae) *and Helinus integrifolius* (Rhamnaceae)*.* Trees and shrubs were the most common plant-life form while leaves were the most popular plant part. Pig fats, red ochre (Luvhundi soil) and ashes were the most cited non-plant materials. These documented natural resources are frequently prepared by crushing and mostly used to heal wounds.

**Conclusion:**

Traditional knowledge concerning folk cosmeceuticals is mostly held by elders. The high number of natural resources documented is an indication that Vhembe district is rich in ethnopharmacological knowledge. Scientific investigation of the efficacies and safety of these natural resources is highly recommended as a drive aimed at innovations with benefits to the rural communities who are the custodians of this valuable knowledge.

## Background

The use of natural resources, especially plant material, for skin diseases and cosmeceutical purposes, is an ancient practice in many cultures globally [1]. Natural resources refer to substances that occur naturally and include plants, animals and micro-organisms. Plant derived-extracts are more common than animal derived-extracts as a source of cosmeceuticals [2]. Despite the continuous neglect of folk cosmeceuticals in favour of the synthetic ones, natural resources are still utilised for skin health in many rural areas [1, 3–5]. The ease of access and belief in the efficacy of indigenous knowledge are common reasons for the continuous dependence on these natural resources.

The bio-compounds from natural resources have been successfully used in skin-care treatment due to their effectiveness and safety. Martins et al. [6] emphasised that the suppliers of the cosmetic industry are embracing the need to include extracts from natural resources because they contain essential vitamins and minerals that exert ultraviolet and anti-oxidant protection and general anti-aging benefits. Recently, the pharmaceutical industry is embracing the ideal of incorporating antioxidants derived from natural resources into their products because they contain chemicals that are valuable in cosmeceuticals [7]. Furthermore, natural antioxidants provide health benefits such as anti-aging, anti-inflammatory, and anti-microbial properties that are suitable for cosmetic purposes [8].

The demand for natural resources for cosmeceuticals is increasing globally. The use of plant-based remedies remains entrenched in the healing practices of developing countries [9]. According to Statistics South Africa [10], South Africans consult both public health facilities and traditional practitioners for rememdies against common illnesses including skin diseases. Even though several ethnobotanical studies have been conducted in Limpopo province, including Vhembe district [11–17], specific attention on natural resources utilised as cosmeceuticals for skin diseases remains understudied. While Mabogo [16] documented 44 plants with cosmeceutical potential, Mahwasane et al. [11] and Magwede et al. [13] recorded 2 and 13 plants, respectively. A recent study by Constant and Tshisikhawe [12] did not highlight any plant with cosmeceutical value. Ndhlovu et al. [17] focused on plants used for cosmetic and cosmeceutical purposes by the Vhavenda women. Thus, the current study aims at documenting natural resources used as folk cosmeceuticals among households in rural communities located in Vhembe district.

## Methods

### Study area

**Fig. 1:**
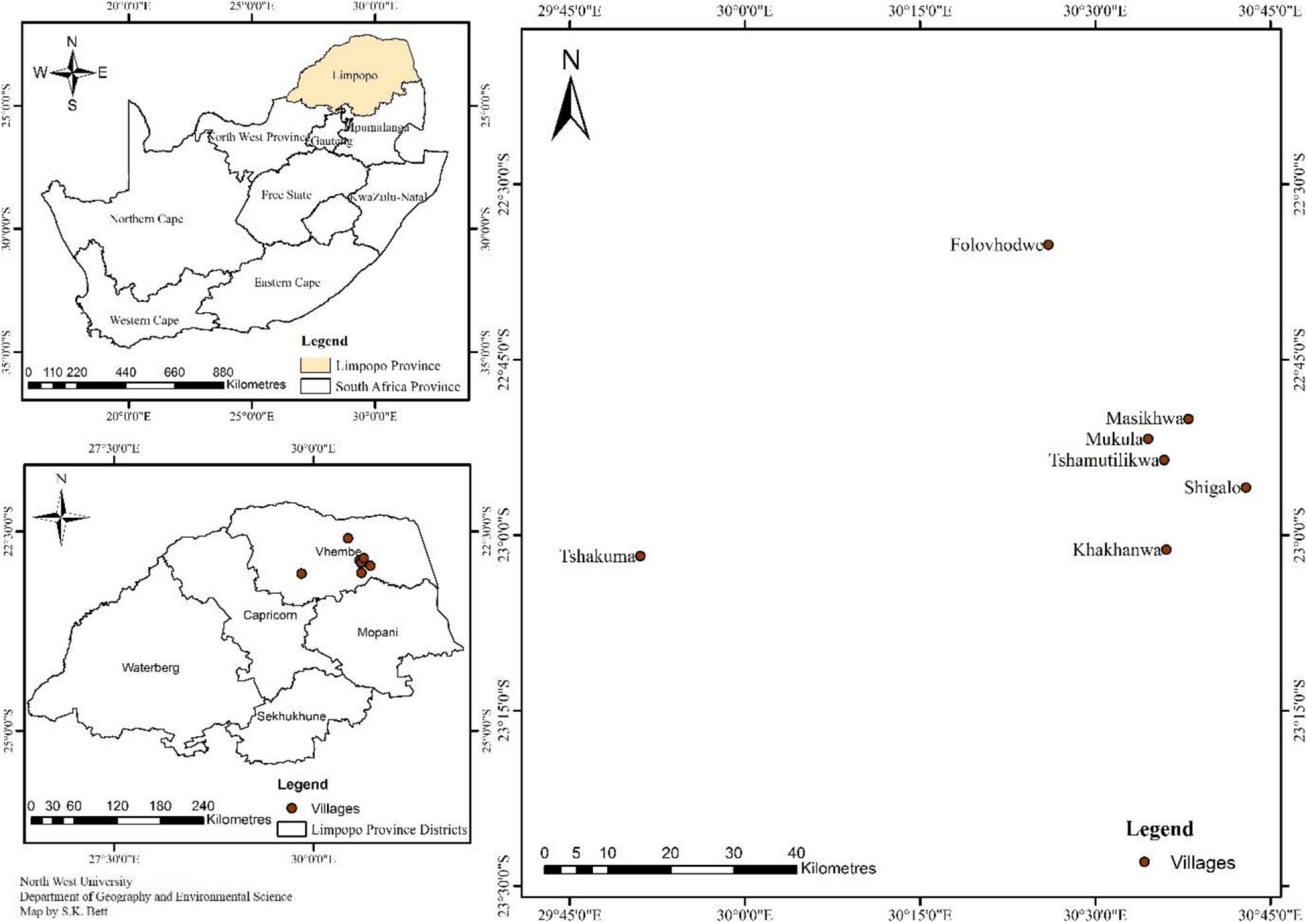
Location of the selected villages in Vhembe district municipality, Limpopo province, South Africa. (Luvhimbi = Masikhwa). The map was drawn by S.K. Bett using ArcMap 10.3.1 software

The study was conducted in six villages situated on the northern side of Limpopo province, South Africa **(**Fig. 1**)**. Vhembe district has a population of 1,393,949 with 382,346 households and mainly dominated by the Vhavenda and VaTsongas [18]. It comprises of four local municipalities namely Thulamela, Collins Chabane, Makhado, and Musina. The district municipality is predominantly rural, with more than 85% of its population living in tribal settlements and farms, and only 5% living in urban areas [19]. Vhembe district is mainly covered with the vegetation of Savannah biome and topography and is characterised by Soutpansberg (“Salt Pan Mountain”).

### Ethnopharmacognostic survey

The survey involved 71 participants who were knowledgeable about natural resources utilised for folk cosmeceuticals. The participants included herbalists, traditional practitioners and laypeople. Convenient sampling was used to get the participants. The households were randomly chosen using every fifth (5th) house in the community because all the possible samples that were chosen came from the population that had the same probability to belong to the sample. The convenient sampling was used by asking questions from household members that were willing to participate. This sampling method was adopted to provide the community members the freedom to choose whether to participate or otherwise. In fact, those who were not interested in participating in the study referred the researcher to the people who were known to have the required knowledge.

Semi-structured questionnaires were used to collect data from the participants. The questionnaire was divided into four categories. Category A entailed the interview log that requested information about the name of the community, date of data collection, local municipality and demography of participants. Category B comprised inquiries about natural resources utilised for folk cosmeceuticals, method of preparations and administration and part used. Category C consisted of indigenous knowledge and practices involved in the formulation and use of folk cosmeceuticals, and lastly Category D comprised questions about the factors influencing the use of folk cosmeceuticals. The interview was conducted in Tshivenda and Xitsonga with the assistance of two translators. Some of the interviews were conducted in an informal gathering, although the original intention of the researcher was to interview one person at a time. Interviewing participants that gathered informally contributed a lot to the study. It was observed that some of the participants would only contribute their knowledge when they were in a group which benefited the researcher in saving time and helping to gather more data.

### Plant collections and identification

Plants were collected both from home gardens and natural vegetation during the fieldwork with the assistance of knowledge holders and translators. The permission to collect the required specimens was granted by the Department of Environmental Affairs, Limpopo Province and the tribal authority. Voucher specimens of the plants were deposited at the herbarium of the South African National Biodiversity Institute (SANBI), Pretoria for identification. Plants were also identified with the relevant books and with the assistance of an expert (Botanist).

### Ethics approval and consent to participate

The current study was approved (ethical clearance no: NWU-07740-17-A9) was by the research ethics committee of North-West University, Mmabatho, South Africa. Permission letter to access and conduct research in six communities was granted by the traditional leaders. Data were collected with full agreement with the participants and consent form was issued to them which clearly stated and explained that the participants were volunteering and the details of the study which included aim, objectives and how data were going to be collected.

### Data analysis

The data analysis was carried out using both descriptive and inferential statistics utilizing percentage and frequency. Data from the questionnaire were analysed using IBM SPSS analytical tool, Microsoft Excel 2016. Ethnobotanical indices such as frequency of mention (F), use-value (UV) and Relative frequency of citation (RFC) were calculated as detailed previously [20].

## Results

### Socio-demographic characteristics of participants

In the current survey, the 71 participants that were interviewed had diverse demographic characteristics (Table 1). Most of the participants involved in the survey were from Shigalo and Tshakuma communities. The majority (76%) of the participants were females and the dominating (35%) age group was those individuals older than 70 years. The majority of the participants belonged to the Venda tribe (66%) and most of the participants lacked formal employment.

**Table 1:** Demographic characteristics of participants in the study area

Demographic features	Frequency	Percentage (%)
***Gender***		
Male Female	17 54	24 76
***Age***		
31–40	9	13
41–50	6	8
51–60	15	21
61–70	16	23
71-above	25	35
***Type of employment***		
Not employed	65	92
Informal	5	7
Formal	1	1
***Monthly income***		
< 500	20	28
500–1500	13	18
1500–3000	38	54
***Tribe***		
Vhavenda	47	66
Tsonga	21	30
Other	3	4
***Villages***		
Tshamutilikwa	7	9.8
Luvhimbi (Masikhwa)	8	11.3
Khakhanwa	3	4.2
Shigalo	22	31
Tshakuma	25	35.2
Folovhodwe	6	8.5

### Plant and non-plant resources used for cosmeceuticals

**Fig. 2:**
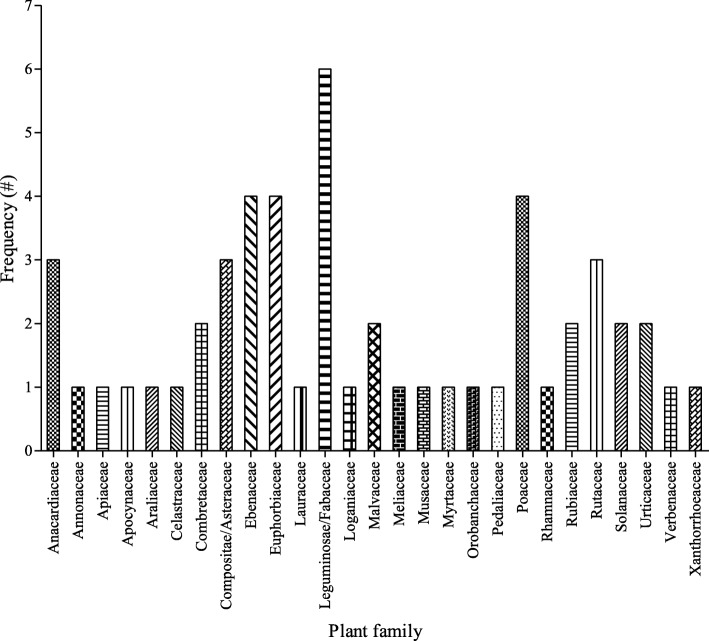
Frequency of plant families used as folk cosmeceuticals among rural communities in Vhembe district municipality, Limpopo province, South Africa

**Fig. 3:**
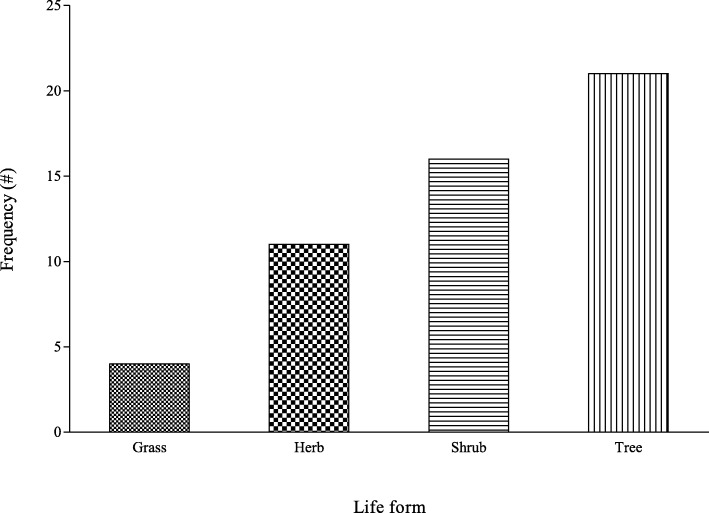
Frequency of plant life-forms used as folk cosmeceuticals among rural communities in Vhembe district municipality, Limpopo province, South Africa

**Fig. 4:**
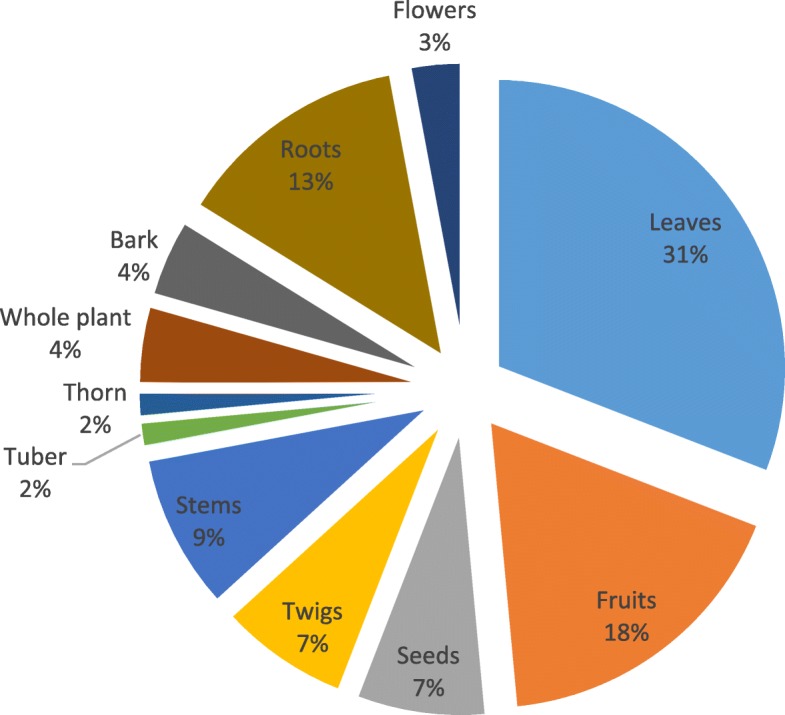
Distribution (%) of plant parts used for preparing folk cosmeceutical remedies among rural communities in Vhembe district municipality, Limpopo province, South Africa, *n* = 68

In total, 52 plants from 27 families were cited as being used as folk cosmeceuticals in the study area (Table 2**;** Fig. 2). As shown in Fig. 2, most of the plants belong to the following families: Leguminosae/Fabaceae (6), Ebenaceae (4), Poaceae (4), Euphorbiaceae (4), Anacardiaceae (3), Compositae/Asteraceae (3) and Rutaceae (3). In terms of frequency, the most cited plants were *Dicerocaryum zanguebarium, Ricinus communis, Helinus integrifolius, Zea mays* and *Annona senegalensis*. Shrubs and trees constituted the most (71%) occurring plant life-forms while the proportion of herbs and grasses were 21 and 8%, respectively **(**Fig. 3**)**. Even though diverse plant parts were used as cosmeceuticals, the leaves (32%), fruit (18%) and roots (13%) were the most utilised plant parts (Fig. 4). Plants with the highest UV were *Aloe vera* (0.084*)*, *Euclea divinorum* (0.084)*, Bauhinia thonningii* (0.070) and *Citrus limon* (0.056) (Table 2).

**Table 2:** Plants used as folk cosmeceuticals among rural communities in Vhembe district municipality, Limpopo province, South Africa

Scientific name (Voucher number)	^**a**^Common name(s)	Family	Part(s) used	^**b**^F	Use-value	^**c**^RFC	Method of preparation and administration	Life-form	^**d**^Location
*Acmella caulirhiza* Delile Syn: *Eclipta filicaulis* Schumach. & Thonn. (MVS 007)	Tshishengeraphofu (V)	Compositae/ Asteraceae	Leaves	2	0.042	0.03	The leaves are crushed and mixed with water to wash wounds to enhance healing. The crushed leaves are infused in water to wash sores. The powder of the crushed leaves is mixed with saliva and applied topically for wound healing	Herb	LVB TKM
*Albizia harveyi* E*.*Fourn*.* (MVS 019)	Molela (X) paperbark false-thorn (E)	Leguminosae/ Fabaceae	Roots	1	0.014	0.01	The roots are burned and applied to remove rash	Tree	SGL
*Aloe vera* (L.) Burm.f. Syn: *Aloe barbadensis* Mill. (MVS 031)	Mhangani (X)/ Tshikopa (V) Aloe (E)	Xanthorrhoeaceae	Leaves	5	0.084	0.07	The liquid from the leaves is applied topically to remove ringworms, moisturize the skin, remove stretch marks, rash and to heal burned skin and wound	Herb	LVB TMTL TKM FLVD SGL
*Annona senegalensis* Pers*.* (MVS 005)	Muembe (V) African Custard-apple (E)	Annonaceae	Twigs	11	0.014	0.15	The twig is crushed and used as a toothbrush to clean the teeth	Shrub	TKM LVB
*Bauhinia thonningii* Schum. Syn: *Piliostigma thonningii* (Schum.) Milne-Redh (MVS 018)	Xidengana/ denga (X) camel’s foot (E)	Leguminosae/ Fabaceae	Fruit	5	0.070	0.07	The fruit is burned and mixed with oil which is applied on ringworms, sores and for treating skin irritation. The liquid from fruit applied to remove pimples and wounds	Shrub	SGL
*Bidens pilosa* L. (MVS 047)	Mushidzhi (V) Black jack (E)	Compositae/ Asteraceae	Leaves Fruit	1	0.038	0.01	The leaves are crushed and the liquid is applied on wounds. The fruit juice is mixed with ashes to remove rash	Herb	LVB
*Citrus limon* (L.) Osbeck (MVS 002)	Tshikavhave (V) Lemon (E)	Rutaceae	Fruit	3	0.056	0.04	Juice from the fruit is applied on the skin for moisturizing, removing wrinkles, scars and pimples	Tree	TKM FLVD SGL
*Citrus reticulata* Blanco (MVS 050)	Swiri (V) Orange (E)	Rutaceae	Fruit	2	0.038	0.03	The fruit juice is applied on the skin to clean and soften it	Tree	LVB
*Combretum imberbe* Wawra (MVS 038)	Mondzo (X) Leadwood (E)	Combretaceae	Bark	1	0.014	0.01	The bark is ground and mixed with water to remove sores by bathing	Tree	SGL
*Cussonia spicata* Thunb (MVS 040)	Musenzhe (V) Cabbage tree (E)	Araliaceae	Leaves	1	0.014	0.01	The leaves are crushed and applied as a paste on ringworms	Tree	TKM
*Dicerocaryum zanguebarium* (Lour.) Merr. (MVS 022)	Dinda (X) /museto (V) Boot protectors (E)	Pedaliaceae	Leaves	61	0.042	0.85	The leaves are mixed with water for bathing; also relaxes hair and removes dandruff	Herb	TKM TMTK LVB KKN FLVD SGL
*Dichrostachys cinerea* (L.) Wight & Arn. Syn*: Acacia cinerea* (L.) Spreng. (MVS 011)	Murenzhe (V) Sickle bush (E)	Leguminosae/ Fabaceae	Fruit Bark	2	0.042	0.03	The fruit is burned and the ashes applied for wound healing. The bark is crushed and mixed with oil to remove ringworms. The bark is boiled and water used to wash the wound	Shrub	TKM KKN
*Diospyros lycioides* Desf. (MVS 025)	Muthala (V) Quilted Bluebush (E)	Ebenaceae	Twigs Fruit	3	0.038	0.04	The twig is crushed to clean the teeth. The fruit is juice is mixed with water to wash wounds	Shrub	TKM LVB
*Diospyros mespiliformis* Hochst. ex A. *DC.* (MVS 017)	Ntoma (X) Musuma (V) Jackal berry (E)	Ebenaceae	Fruit Leaves Twigs	5	0.042	0.07	Liquid from the leaves and fruit are applied on skin for eradicating ringworms. The twig is crushed to clean the teeth	Tree	TMTK TKM KKN SGL
*Diospyros natalensis* (Harv.) Brenan (MVS 043)	Xintomatomane (X) Acorn Jackal-berry (E)	Ebenaceae	Twigs	3	0.014	0.04	The tip of twig is crushed and used to clean the teeth	Tree	SGL
*Dombeya rotundifolia (*Hochst.) Planch (MVS 036)	Tshiluvhari (V) Wild Pear/plum (E)	Malvaceae	Leaves	1	0.038	0.01	The leaves are crushed and mixed with water to wash and dye hair	Tree	KKN
*Euclea divinorum* Hiern (MVS 013)	Nhlangula (X) /mutangule (V) Magic guarri (E)	Ebenaceae	Leaves	7	0.084	0.10	The crushed leaves are mixed with water or the leaves are boiled and used for bathing to remove skin irritation, ringworms, rash, pimples, chickenpox	Shrub	LVB KKN TKM SGL
*Eugenia capensis* subsp. *natalitia* (Sond.) F.White Syn: *Eugenia natalitia* Sond (MVS 001)	Tshitanzwa-tanzwane (V) Forest Myrtle (E)	Myrtaceae	Roots	1	0.014	0.01	The roots are ground and soaked in water to wash sores.	Shrub	TMTK
*Gardenia volkensii* K. Schum. (MVS 020)	Tshiralala (V) bushveld gardenia (E)	Rubiaceae	Flowers	1	0.014	0.01	The flower is mixed with water to bathe for controlling odour	Tree	FLVD
*Helinus integrifolius* (Lam.) Kuntze (MVS 032)	Mpupungwa/ mugumwa (V) Soap Bush/Plant (E)	Rhamnaceae	Leaves	13	0.038	0.18	The leaves are mixed with water to make foam to bathe and wash hair.	Shrub	TKM TMTK
*Heteromorpha arborescens* (Spreng.) Cham. & Schltdl. (MVS 008)	Muthathavhanna (V) Parsley tree (E)	Apiaceae	Leaves	1	0.014	0.01	The leaves are squashed and applied as a paste for healing burned skin	Tree	LVB
*Hyperacanthus amoenus* (Sims) Bridson (MVS 049)	Murombe (V) Spiny-gardenia (E)	Rubiaceae	Fruit	1	0.014	0.01	The fruit called *thomba liquid,* it is applied on the skin for removing pimples	Shrub	TKM
*Indigofera arrecta* Hochst. A. Rich (MVS 034)	Muswiswa (V) Java indigo (E)	Leguminosae/ Fabaceae	Twigs Leaves	2	0.042	0.03	The twig is crushed and used to clean the teeth. The tip of the twig is crushed and is dipped in ashes to clean teeth The leaves are infused in water for bathing	Herb	TKM
*Jatropha curcas* L*.* Syn: *Castiglionia lobata* Ruiz & Pav. (MVS 004)	Mupfure donga (V) Barbados nut Bubblebush (E)	Euphorbiaceae	Stem Leaves Roots	6	0.038	0.08	Liquid from crushed leaves and stems are applied to moisturize the skin. The roots are ground and soaked in water to wash wounds	Shrub	TMTK TKM
*Jatropha zeyheri* Sond*.* Syn: *Jatropha brachyadenia* Pax & K.Hoffm. (MVS 052)	Xidemeja (X)	Euphorbiaceae	Leaves	1	0.014	0.01	The leaves are squashed and applied as a paste for wound healing	Herb	SGL
*Lannea schweinfurthii* var. *stuhlmannii* (Engl.) Kokwaro Syn: *Lannea stuhlmannii* Engl. *(*MVS 039)	Ndivata (X) False marula (E)	Anacardiaceae	Leaves	1	0.014	0.01	The leaves are crushed and smeared on wounds for healing	Tree	SGL
*Lippia javanica* (Burm. f.) Spreng (MVS 041)	Musudzungwane (V) Fever tea (E)	Verbenaceae	Leaves	4	0.038	0.05	The leaves are rubbed on the skin for the treatment of rash. The leaves are crushed and rubbed with oil on the body for the treatment of rash.	Shrub	TKM TMTK KKN
*Musa acuminata* Colla (MVS 028)	Muova (V) Banana (E)	Musaceae	Flowers Leaves	4	0.038	0.05	Liquid from leaves and flower are applied for treating wounds and burned skin	Tree	TKM LVB
*Obetia tenax* Friis Syn*: Urera tenax* N.E. Br. (MVS 006)	Thanga (V) Rock tree (E)	Urticaceae	Seeds	2	0.014	0.03	The seeds are ground to moisturize the skin	Tree	TMTK FLVD
*Peltophorum africanum* Sond. (MVS 012)	Musese (V) African Black Wattle (E)	Leguminosae/ Fabaceae	Bark	1	0.014	0.01	The bark is boiled and drunk to heal mouth sores	Tree	TMTK
*Persea americana* Mill*.* (MVS 052)	Afukhada (V) Avocado (E)	Lauraceae	Seeds Fruit	6	0.042	0.08	The seeds are crushed and used as face-wash to remove blackheads on the skin. The fruit is rubbed on the skin to moisturize and soften it	Tree	TKM KKN FLVD
*Phragmites mauritianus* Kunth (MVS 014)	Lutanga (V) Lowveld Reed (E)	Poaceae	Thorns whole plant	3	0.038	0.04	The thorn is used to prickle and remove moles. The whole plant is burned and the ashes applied for removal of stretch marks.	Grass	TMTK TKM
*Pouzolzia mixta* Solms Syn*: Pouzolzia huillensis* Hiern (MVS 009)	Muthanzwa (V) Snuggle-leaf (E)	Urticaceae	Roots	1	0.014	0.01	The roots are crushed to powder and applied on a wound to heal it.	Shrub	TMTK
*Ricinus communis* L. (MVS 016)	Nhlampfurha (X) mupfure (V) Castor oil plant	Euphorbiaceae	Seeds	20	0.042	0.28	Oil from the fried seeds are used for moisturizing the skin and hair	Shrub	TKM TMTK LVB FLVD SGL
*Salacia rehmannii* Schinz. (MVS 026)	Phathatsimima (V)	Celastraceae	Roots	1	0.014	0.01	The roots are ground and mixed with water to wash sores	Shrub	TMTK
*Sclerocarya birrea* (A.Rich.) Hochst. (MVS 033)	Murula (V) Marula (E)	Anacardiaceae	Stem Seeds	2	0.042	0.03	The stem is burned and applied to wounds. The seeds are ground and mixed with water till it becomes soft to moisturize the skin and as anti-aging	Tree	LVB FLVD
*Searsia lancea* (L.f.) F.A.Barkley (MVS 045)	Mushakaladzane (V) Karee (E)	Anacardiaceae	Leaves	5	0.042	0.07	The leaves are crushed and mixed with water to clean the skin and to treat rashes. The leaves are boiled in water to bathe to remove pimples	Tree	LVB KKN FLVD
*Senna occidentalis* (L.) Link Syn: *Cassia occidentalis* L. (MVS 037)	Nembenembe (X) Munembenembe (V) Coffee senna (E)	Leguminosae/ Fabaceae	Leaves	2	0.038	0.03	The leaves are crushed and made as a paste to apply on burned skin and for wound healing	Shrub	LVB SGL
*Setaria acromelaena (*Hochst.) T.Durand & Schinz (MVS 046)	Xihovane (X) Bristle Grass (E)	Poaceae	Stem	1	0.014	0.01	The stem is crushed and infused with water to remove sores.	Grass	SGL
*Sida cordifolia* L. (MVS	Mutudo (V) Flannel Weed (E)	Malvaceae	Roots	1	0.014	0.01	The roots are burned and the ashes are applied for wound healing	Herb	TKM TMTK
*Solanum panduraeforme* Dunal (MVS 035)	Mututulwa (V) Apple of Sodom (E)	Solanaceae	Fruit	2	0.042	0.03	The fruit is burned and applied after incision on ringworms. The liquid is applied to heal a wound and chickenpox.	Herb	TKM
*Solanum tuberosum* L. (MVS 048)	Potato (E)	Solanaceae	Tuber	1	0.014	0.01	The peels of potatoes are rubbed on rash for their removal	Herb	SGL
*Striga asiatica* (L.) Kuntze (MVS 044)	Vhuri (V) Red witchweed (E)	Orobanchaceae	Whole plant	2	0.014	0.03	The plant is burned and applied for healing of a wound.	Herb	TMTK
*Strychnos spinosa* Lam. (MVS 023)	Muramba (V) Spiny monkey orange	Loganiaceae	Fruit	1	0.014	0.01	Fruit juice is applied on ringworms for their removal	Tree	LVB FLVD
*Synadenium cupulare* (Boiss.) L.C. Wheeler (MVS 042)	Muswoswo (V) Crying Tree (E)	Euphorbiaceae	Stem	2	0.014	0.03	Liquid from the cut stem is applied on the skin for the removal of moles	Shrub	TKM
*Tabernaemontana elegans* Stapf (MVS 029)	Muhatu (V) Toad tree (E)	Apocynaceae	Stem Roots	2	0.038	0.03	Liquid from the cut stem is applied to the skin for the removal of ringworms. The roots are burned, ground and applied on the skin for the removal of ringworms	Shrub	LVB
*Terminalia sericea* Burch, ex DC (MVS 015)	Nkonono (X) mususu (E) silver cluster-leaf (E)	Combretaceae	Roots Leaves	3	0.038	0.04	The roots are burned and applied on the skin for the removal of pimples. The leaves are crushed and mixed with oil to moisturize the skin	Tree	TMTK SGL
*Trichilia emetica* Vahl (MVS 027)	Nkuhlu (X) Natal mahogany (E)	Meliaceae	Seeds	1	0.014	0.01	The seeds are ground to produce oil for moisturizing the skin	Tree	SGL
Unidentified plant species (MVS 003)	Grass (E)	Poaceae	Whole plant	4	0.038	0.05	The grass is burned and the ashes are applied for healing of sores and wounds.	Grass	SGL
*Vernonia fastigiata* Oliv. & Hiern (MVS 021)	Tanyi (V) Narrow-leaved Vernonia (E)	Compositae/ Asteraceae	Leaves	2	0.014	0.03	The leaves are crushed and smoothly rubbed on the skin for the removal of wound scars.	Herb	TMTK
*Zanthoxylum davyi* Waterm. (MVS 024)	Munungu (V) Fever Tree Knobwood (E)	Rutaceae	Roots Leaves Stem	4	0.038	0.05	The roots are ground and the leaves crushed and applied for wound healing. The crushed stem is used to wash the teeth.	Tree	TMTK LVB
*Zea mays* L. (MVS 48)	Mufhumbu ha mavhele (V) Maize (E)	Poaceae	Fruit	12	0.038	0.16	Cob is ground and mixed with water and used for bathing in order to treat rash. Cob is ground and applied directly to pimples.	Grass	TKM LVB TMTK KKN

^a^Common name: V = Venda, E = English, X = Xitsonga; ^b^F = Frequency; ^c^RFC = Relative frequency citation

^d^Location: *TKM* Tshakuma, *TMTK* Tshamutilikwa, *LVB* Luvhimbi (Masikhwa), *SGL* Shigalo, *KKN* Khakhanwa, *FLVD* Folovhodwe

Twenty-two (22) non-plants materials were recorded as folk cosmeceuticals among rural communities (Table 3). In terms of frequency, the most common (with more than 10 citations) non-plant materials were *nguluvhe/honje* (pig fats), *luvhundi* (soil), *mafhura tharu/ntlharo* (python fat) and wood ashes/coal. In addition, nguluvhe/honje, wood, ashes/coal, soil and sandy soil had the highest (0.042–0.056) UV among the non-plant resources (Table 3).

**Table 3:** Non-plant resources used as cosmeceuticals in Vhembe district municipality, Limpopo province, South Africa

Local/common name(s)	Type of natural resource	^**a**^F	Use-value	^**b**^RFC	Method(s) of preparation and administration	^**c**^Location(s)
Cattle fat	Cattle fat	9	0.014	0.12	The milk is fermented and boiled to make oil which is applied as a skin moisturiser.	Tshakuma Luvhimbi Khakhanwa Tshamutilikwa Folovhodwe Shigalo
Cow dung	Manure	3	0.038	0.04	The moist manure is applied on the feet and washed with water to remove cracks. Manure is also mixed with menstruation blood to remove silver stripes.	Tshakuma
Cow milk	Milk	5	0.014	0.07	The milk is cooked to make a cream and applied topically as a skin moisturiser.	Tshakuma Khakhanwa
Goat	Manure	1	0.014	0.01	The manure is burned and applied for the removal of rash	Khakhanwa
Green algae of river (Hololo)	Fungi	3	0.038	0.04	Hololo is burned and mixed with leaves of musuma to treat ringworms. The algae are burned and applied on wounds for healing	Luvhimbi
Khedi	Soil	1	0.014	0.01	Soil particles are ground to a fine powder and applied on the face for enhancing the complexion	Tshakuma
Lizard manure	Manure	1	0.014	0.01	The manure is crushed and applied directly to a wound for healing.	Tshamutilikwa
Luvhundi soil	Soil	17	0.042	0.23	The soil is mixed with oil or water and applied on the feet to remove cracks. It is also applied as a sun protector. Soil is mixed with oil to remove rash.	Tshakuma Luvhimbi Khakhanwa Folovhodwe Shigalo
Mafhura tharu/ntlharo	Python fat	13	0.042	0.18	Python fat is extracted by frying the python and the oil is applied on burned skin and wounds for healing and removal of scars.	Tshakuma Tshamutilikwa Khakhanwa Folovhodwe
Marumbuda White/green substance on stones usually after heavy rain	Fungi	1	0.042	0.01	Applied to the skin for the removal of stretchmarks, ringworms and rash.	Tshakuma Folovhodwe
Mtaba (sand)	Soil	1	0.014	0.01	The soil is rubbed on the teeth for cleansing.	Tshakuma
Munyaka soil	Yellow and brown soil	2	0.014	0.03	The soil is applied directly to the face as a make-up foundation.	Khakhanwa
Munyaka stone	Stone	2	0.014	0.01	The stone is crushed to a fine powder and mixed with water to soften the skin.	Luvhimbi
Ngulube daka	Oil/fat	1	0.014	0.01	Pig fat is applied as skin moisturizer.	Tshakuma
Nguluvhe/Honje	Pig fat	50	0.056	0.70	The oil is extracted by cooking and used as a skin moisturiser and to remove cracks. Also used as soap to soften and protect the skin.	Tshakuma Tshamutilikwa Luvhimbi Khakhanwa Shigalo Folovhodwe
Salt water/sea water	Water	2	0.038	0.03	Salt and seawater are used to remove rash and ringworms.	Shigalo
Sandy Soil	Soil	11	0.014	0.15	The sandy soil is rubbed on the teeth for cleansing. It is mixed with water and rubbed on the skin for the removal of dirt	Tshakuma Luvhimbi Folovhodwe Shigalo
Stone	Stone	18	0.038	0.25	The stone is used to scrub the feet to remove cracks and dirt removal.	Tshakuma Luvhimbi Khakhanwa Folovhodwe Shigalo
Tshinyai	Soot	3	0.038	0.04	Soot is mixed with oil and applied on the skin to remove rash and stretchmark.	Tshakuma Luvhimbi Tshamutilikwa Shigalo
Urine	Urine	3	0.014	0.04	The urine is applied to burned skin for healing.	Tshakuma Shigalo
White mountain stone	Stone	5	0.038	0.07	The stone is ground into powder form and rubbed on the teeth for cleaning and whitening.	Tshakuma Shigalo
Wood ashes/coal	Ashes	11	0.056	0.15	The wood coal is ground and rubbed on the teeth for cleansing. It is also applied around the eyes as make-up. The wood coal is ground and mixed with oil to dye and soften the hair.	Tshakuma Luvhimbi Folovhodwe Shigalo

^a^*F* Frequency, ^b^*RFC* Relative frequency citation, ^c^Location: Luvhimbi = Masikhwa (on the map in Fig. 1)

### Mode of preparation and administration of folk cosmeceuticals

**Fig. 5:**
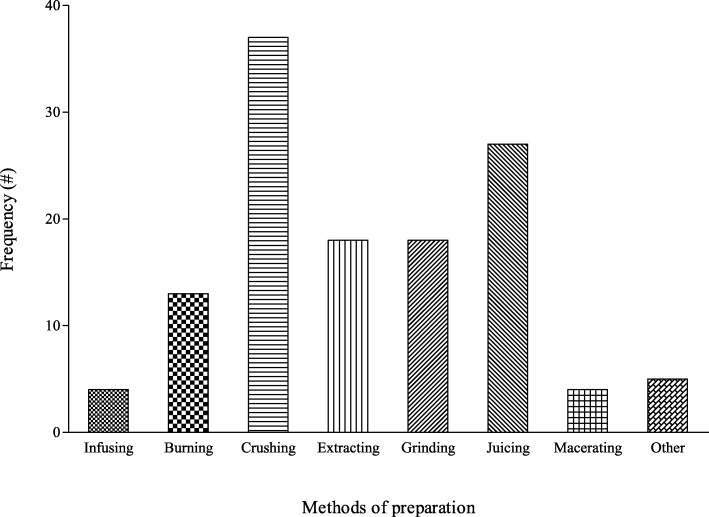
Frequency of the preparation methods used as folk cosmeceuticals among rural communities in Vhembe district municipality, Limpopo province, South Africa

The natural resources used as folk cosmeceuticals were prepared by diverse means such as infusion, grinding and maceration (Fig. 5). The most common methods of preparation of non-plant natural resources were crushing (33%), while maceration (3.5%) and infusion (3.5%) were the least common methods. These natural resources used for folk cosmeceuticals are applied in the form of powder, poultice, juice and infusion. The majority (88%) of the natural product remedies were applied topically. In terms of the broad categories, the remedies were administered for skin afflictions, cosmetics, anti-oxidants and hair care (Table 4).

**Table 4:** Frequency of citations for natural resources used for skin conditions in Vhembe district municipality, Limpopo province, South Africa

Category	Use	Number of natural resource
Skin afflictions	Wounds	27
	Burn	7
	Ringworms	12
	Moles	2
	Pimples	8
	Chickenpox	1
	Rash	13
	Cracks on feet	6
	Stretchmark	4
	Skin irritation	3
	Scar removers	2
	Sores	12
Cosmetic uses	Teeth hygiene	11
	Soaps	6
	Facial scrubs and mask	3
	Body creams	16
Hair care	Shampoo and dye	6
Antioxidants	Skin protection	3
	Wrinkles removal	1
	Skin softening	6

### Indigenous knowledge and practices of folk cosmeceuticals

As highlighted by some of the participants, skin diseases are believed to have an underlying spiritual cause. For instance, some skin diseases are believed to be caused by disobeying ancestors, result from misbehaving in a community and evil spells. As a result, rituals are often recommended and performed for the healing of the skin afflictions. Healers are known to perform ‘*Gumululo*’ which is a ritual used to remove sores from infected skin. Traditional practitioners wash the sores by sprinkling water mixed with the unspecified concoction. Some severe skin diseases require the patient to stay in an isolated area for a certain duration in order to be healed. When it is time for the patient to return home, the family members are given a similar concoction to prevent the same disease. In some instances, patients wash in a lake called ‘*Dzivha la fundudzi*’ in Venda because it is a sacred river, a river of gods. For them to access the lake to wash away the disease, permission is requested from the priest and traditional practitioner that guard the lake.

## Discussion

As emphasized by Fongnzossie et al. [5], there is inadequate documentation of the ethnobotanical knowledge on cosmeceuticals. Furthermore, Lall and Kishore [21] highlighted the existing research gaps that involve both the inadequacies in ethnobotanical documentation and scientific evaluation of the plants used skincare in South Africa. The current ethnopharmacognostic survey indicated that the custodians of knowledge on natural resources with cosmeceutical potential were females and elders (above 70 years). Indigenous knowledge often held by the elders in the communities is transmitted orally from generation to generation but its practice seems to be declining due to the lack of interest by the youth [22].

### Diversity of natural resources used for folk cosmeceuticals

The high quantity of natural resources as well as the diversity of flora identified, is an indication that the study area has rich indigenous knowledge on folk cosmeceuticals. As an addition to existing surveys in Vhembe district [11–17], the current study documented new plants (for e.g. *Zea mays, Eugenia natalitia, Salacia rehmannii)* for the first time, as natural resources for folk cosmeceuticals. Some of these plants were previously documented as medicine without the details of the diseases in recently study by Magwede et al. [13]. The current study indicated that the natural resources used to treat the same skin problems differ among the rural communities. It was also evident that a single plant is utilised for more than one skin problem; and a single skin problem has more than one natural resource as the recommended remedies.

Some of the plants were used in almost all of the rural communities where the survey was conducted. These plants included *Dicerocaryum zanguebarium* (Museto), *Ricinus communis* (Nhlampfurha/mupfure), *Zea mays* (mavhele)*, Euclea divinorum* (Nhlangula/mutangule) and *Diospyros mespiliformis* (Musuma)*.* These commonly used plants have been recorded by other authors, which is an indication of their cosmeceutical value. For instance, Chigora et al. [23] stated that the whole plant of *Dicerocaryum zanguebarium* (Museto) is used to make foam which is inserted into the vagina to dilate the birth canal, in Zimbabwe. The juice from the plant is used as shampoo by people of Gazankulu in Limpopo province [24]. The current study affirmed the use of *Dicerocaryum zanguebarium,* as the participants indicated the use of the whole plant to wash their hair. Many surveys have identified and documented *Ricinus communis,* [25–28], which suggests that the plant has high medicinal and cosmeceutical values. According to Maroyi [29], the roots of *Ricinus communis* are used indigenously to clean the teeth and to heal tooth-ache. The seeds are used as oil which is applied on sore eyes in Zimbabwe. The leaves are burned, seeds and bark are pulverised and applied as poultices to relieve soreness and inflammation [30]. Similarly, the leaves are mixed with water to wash and cure boils on the skin by the Xhosa people in Eastern Cape, South Africa [31]. The different plant parts used from the same plant for different cosmeceutical purposes is evidence of the variety of indigenous knowledge possessed among different communities and ethnic groups.

The commonly used non-plant resources included wood ashes, pig fat, stones, ochre (Luvhundi soil) and soot (Tshinyae). Ochre is commonly used in initiation schools in South Africa. As stated by the participants, it is used for skin protection from sun and insect bites. The cosmeceutical value of ochre has been recorded in several studies [32, 33]. In Western Sahara, Volpato et al. [34] indicated that Sahrawi refugees apply red ochre around their eyes to reduce solar radiance. The participants mentioned that the wood ashes are utilised to dye hair and make it soft, darken their eyebrows as make-up and for teeth whitening. As part of the remedies for healing skin diseases in the inland Marches, Central-Eastern Italy, Pieroni et al., (2004) indicated that the mixture of ashes and water is used to soften the hair. As indicated by Zhang et al. [35], the people of Bulang, China used soot to blacken their teeth to ensure health of the teeth.

### Distribution of plant families, life-forms and plant parts

The documented plants belong to 27 families and the dominant family was Fabaceae. This plant family was also the most dominant among the study areas in South West Nigeria [36]. On the other hand, Afolayan et al. [37] indicated that Fabaceae is the third most common family after Solanaceae and Asteraceae, among plant families used by the Xhosas for skin diseases. Generally, the Fabaceae is regarded as one of the families with diverse economic and medicinal value [38]. The life-form that dominated was woody plants (trees and shrubs) and most of the remedies were prepared using the leaves. Similarly, high utilisation of the leaves was recorded as folklore phytocosmetics among the communities of South West Nigeria [36]. The use of leaves as folk cosmeceutical emboldens conservation practices, unlike using roots and bark which may cause the death of plants when done indiscriminately. According to Mathabe et al. [39], remedies were commonly prepared from bark collected at any time and though, in some instances, some plants were not collected because the formulation requires the use of the roots be collected.

### Cosmeceutical applications, method of preparation and administration

Skin-related diseases are diverse and occur without any discrimination relating to the age, gender or social status of an individual. As shown in Table 4, the highest cosmeceutical application mentioned by the participants was wounds (27) and body creams (16). The current study revealed that natural resources are popular remedies for diverse skin diseases among the local communities. Thus, ethnopharmacological information from surveys remains a valuable source to explore for potential cosmeceutical products that may possess commercial value [40].

The natural resources are prepared as single component remedies or in combination with other natural resources. The study shows that both plants and non-plants are mostly mixed with water and oil to enhance their penetration across the skin layers. The non-plant materials such as urine and cow dung are mixed with the other plant materials to potentiate its effect [15]. Crushing was the most dominating preparation method and these remedies are mostly applied topically as pastes directly on the skin. Based on existing literature on medicinal plants used for skin problems, the method of administrations includes powder, paste, ointment, poultice and infusion [25, 37, 41, 42]. These aforementioned studies indicated that the majority of administration methods are similar to the current study. Mongalo and Makhafola [14] investigated the ethno-botanical knowledge of laymen in Blouberg (Pedi tribe), Limpopo and indicated that the medicinal plants are applied topically on the skin while others are used to wash and rinse the infected body part(s).

### Potential of documented plants with high use-value

Plants with high UV included *Aloe vera* and *Euclea divinorum*, *Bauhinia thonningii* and *Citrus limon*. These aforementioned plants are known for their diverse therapeutic values. *Aloe vera* is an ancient medicinal plant that is used externally for different skin problems. In the current study, the gel of the plant is used to moisturise the skin, remove stretch marks, wounds, ringworm and rash as well as to heal burned skin. Likewise in India, Bhowmik [43] indicated that *Aloe vera* is a ‘*miracle*’ plant with different cosmeceutical applications (to heal cuts, burns, eczema, inflammation, sunburns and as hair styling gel). Furthermore, *Aloe vera* is used in many natural products for the skin which include make-up, moisturisers, soaps, sunscreens, shampoos and lotions [43]. *Aloe vera* is known to be involved in a coordinated cascade of cellular and molecular events that interact with re-epithelialisation and reconstitution processes of the tissue to ensure wound healing [44]. As reviewed by Amoo et al. [45], *Aloe* species are generally used for skin ailments because they exert pharmacological properties such as antimicrobial, anti-inflammatory, and antioxidant activities.

*Euclea divinorum* is a traditional medicine that is used for several ailments, including skin diseases. The current findings also indicated that the plant removes skin irritation, ringworms, rash, pimples and chickenpox. Woldemedhin et al. [46] affirmed the healing property of *Euclea divinorum* by revealing its usefulness against skin ailments such as inflammation of the skin, eczema and scabies in Ethiopia. A study by Otang and Afolayan [47] affirmed the therapeutic value of *Citrus limon* for skin diseases based on the antimicrobial efficacy against a panel of microbes. In the Amathole District, Eastern Cape, South Africa, *Citrus limon* is utilized for diverse purposes. For instance, it is used to reduce skin itching, for skin nourishment, and the pulp left after extraction of the juice is used for the treatment of pimples and wrinkles and to soften facial skin [48]. Similar uses were also recorded in the current study whereby the participants indicated that *Citrus limon* is used for moisturising the skin, removing wrinkles, scars and pimples. Furthermore, Otang and Afolayan [47] demonstrated that *Citrus limon* has high anti-oxidant property which is relevant to the effective treatment of skin ailments.

### Indigenous knowledge and practices on folk cosmeceuticals used against skin diseases

According to Rankoana et al. [49], some of the indigenous aetiology of disease are ancestral spirits, witches and sorcerers. Shenefelt and Shenefelt [50] affirmed that since ancient times, skin diseases have had spiritual and religious origins. There are several ways that Africans explain and understand the causes of diseases. For instance, evil spirits and disobedience to ancestors are believed to cause different diseases [51, 52]. Good-health and well-being is usually understood in terms of the relationship with one’s ancestors and as a result of good behaviour, i.e. if one lives in accordance with the values and norms of the traditions of the community [51]. Quave et al. [53] affirmed that if diseases are observed as superstitious or spiritualistic, they are treated differently. Some of the manifestations of skin inflammation are believed to be caused by wind illness or dead fire illness which requires the performance of rituals accompanied by natural resources [53]. Hence, Reyes-García [54] asserted that the choice of treatment is often related to an understanding of the cause of disease in African indigenous health systems. African indigenous healing system is addressed via two perspectives, which are the spiritual and physical perspectives [51]. Ethnomedicine practices form the basis of indigenous healthcare across different ethnic groups globally [49, 55–57]. The participants stated that their knowledge about folk cosmeceuticals comes from their ancestors, hence, they find it necessary to trust it, otherwise, they will lose their culture and which ultimately will dishonour their ancestors. Furthermore, they mentioned that traditional practitioners use folk cosmeceuticals to honour and obey ancestors. The participants consult traditional practitioners or use folk cosmeceuticals before using western medicine or consulting western practitioners. Some skin diseases cannot be healed by applying conventional cosmeceuticals, hence, indigenous people use folk cosmeceuticals because they believe that folk cosmeceuticals have spiritual modes of action that ensures effectiveness.

## Conclusions

This explorative study documented the natural resources (plant and non-plant materials) used for folk cosmeceuticals by six rural communities in Vhembe district municipality, Limpopo province, South Africa. The study identified the natural resources used as folk cosmeceuticals in Vhembe district for future investigation for potential solutions to dermatological problems. In total, 52 plants and 22 non-plant materials were recorded as folk cosmeceuticals. The high number of natural resources is an indication that the area of study is rich in folk cosmeceuticals. As commonly observed with many ethnobotanical surveys, most of the participants were elders. This is an indication that a great deal of effort is needed to document the folk knowledge. However, scientific investigations in terms of the efficacy of natural resources is strongly recommended. For example, toxicity assay of the natural resources will provide insight and understanding of the safety of the folk cosmeceuticals, which may guarantee the safety of local users.

## Abbreviations

FC: Frequency of citation
FLVD: Folovhodwe
KKN: Khakhanwa
LVB: Luvhimbi
RFC: Relative frequency of citation
SANBI: South African national biodiversity institute
SGL: Shigalo
TKM: Tshakuma
TMTK: Tshamutilikwa
UV: Use-value

## References

[1] Pieroni Andrea, Quave Cassandra L, Villanelli Maria Lorena, Mangino Paola, Sabbatini Giulia, Santini Luigina, Boccetti Tamara, Profili Monica, Ciccioli Tamara, Rampa Loredana Giovanna, Antonini Giovanna, Girolamini Claudia, Cecchi Marcello, Tomasi Marco (2004). Ethnopharmacognostic survey on the natural ingredients used in folk cosmetics, cosmeceuticals and remedies for healing skin diseases in the inland Marches, Central-Eastern Italy. Journal of Ethnopharmacology.

[2] Aburjai T, Natsheh FM (2003). Plants used in cosmetics. Phytother Res.

[3] Mahomoodally MF, Ramjuttun P (2016). A quantitative ethnobotanical survey of phytocosmetics used in the tropical island of Mauritius. J Ethnopharmacol.

[4] De Wet H, Nciki S, van Vuuren SF (2013). Medicinal plants used for the treatment of various skin disorders by a rural community in northern Maputaland. S Afr J Ethnobiol Ethnomed.

[5] Fongnzossie EF, Tize Z, Fogang Nde PJ, Nyangono Biyegue CF, Bouelet Ntsama IS, Dibong SD, Nkongmeneck BA (2017). Ethnobotany and pharmacognostic perspective of plant species used as traditional cosmetics and cosmeceuticals among the Gbaya ethnic group in eastern Cameroon. S Afr J Bot.

[6] Martins A, Vieira H, Gaspar H, Santos S (2014). Marketed marine natural products in the pharmaceutical and Cosmeceutical industries: tips for success. Marine Drugs.

[7] Loh Teng-Hern T, Kok-Gan C, Chim Kei C, Tahir Mehmood K, Learn-Han L, Bey-Hing G. Antioxidative potential of a *Streptomyces* sp. MUM292 isolated from mangrove soil. Biomed Res Int. 2018;2018:1–13. 10.1155/2018/4823126 PMC5899857 29805975

[8] Costa R, Santos L (2017). Delivery systems for cosmetics - from manufacturing to the skin of natural antioxidants. Powder Technol.

[9] Makunga NP, Philander LE, Smith M (2008). Current perspectives on an emerging formal natural products sector in South Africa. J Ethnopharmacol.

[10] Statistics-South Africa (Stats SA): General Household Survey; 2017.

[11] Mahwasane ST, Middleton L, Boaduo N (2013). An ethnobotanical survey of indigenous knowledge on medicinal plants used by the traditional healers of the Lwamondo area, Limpopo province, South Africa. S Afr J Bot.

[12] Constant NL, Tshisikhawe MP (2018). Hierarchies of knowledge: ethnobotanical knowledge, practices and beliefs of the Vhavenda in South Africa for biodiversity conservation. J Ethnobiol Ethnomed.

[13] Magwede K., van Wyk B.-E., van Wyk A.E. (2019). An inventory of Vhavenḓa useful plants. South African Journal of Botany.

[14] Mongalo NI, Makhafola TJ (2018). Ethnobotanical knowledge of the lay people of Blouberg area (Pedi tribe), Limpopo Province. S Afr J Ethnobiol Ethnomed.

[15] Arnold H-J, Gulumian M (1984). Pharmacopoeia of traditional medicine in Venda. J Ethnopharmacol.

[16] Mabogo DEN. The ethnobotany of the Vhavenda (Doctoral dissertation, University of Pretoria). Pretoria. 1990.

[17] Ndhlovu PT, Mooki O, Otang Mbeng W, Aremu AO (2019). Plant species used for cosmetic and cosmeceutical purposes by the Vhavenda women in Vhembe District municipality, Limpopo, South Africa. S Afr J Bot.

[18] Stats-SA (2016). Community Survey 2016.

[19] Massyn N, English R, McCracken P, Ndlovu N, Gerritsen A, Bradshaw D, Groenewald P. Disease profile for Vhembe Health District Limpopo. Durban: Health Systems Trust; 2015.

[20] Mwinga J.L., Makhaga N.S., Aremu A.O., Otang-Mbeng W. (2019). Botanicals used for cosmetic purposes by Xhosa women in the Eastern Cape, South Africa. South African Journal of Botany.

[21] Lall N, Kishore N (2014). Are plants used for skin care in South Africa fully explored?. J Ethnopharmacol.

[22] Motlhanka DMT, Nthoiwa GP (2013). Ethnobotanical survey of medicinal plants of Tswapong north, in eastern Botswana: a case of plants from Mosweu and Seolwane villages. Eur J Med Plants.

[23] Chigora P, Masocha R, Mutenheri F. The role of indigenous medicinal knowledge (IMK) in the treatment of ailments in rural Zimbabwe: the case of Mutirikwi communal lands. J Sustainable Dev Afr. 2007;9:26–43.

[24] Liengme CA. Plants used by the Tsonga people of Gazankulu. 1981;13:18.

[25] Abbasi AM, Khan MA, Ahmad M, Zafar M, Jahan S, Sultana S (2010). Ethnopharmacological application of medicinal plants to cure skin diseases and in folk cosmetics among the tribal communities of North-West Frontier Province, Pakistan. J Ethnopharmacol.

[26] Ilavarasan Raju, Mallika Moni, Venkataraman Subramanian (2006). Anti-inflammatory and free radical scavenging activity of Ricinus communis root extract. Journal of Ethnopharmacology.

[27] Scarpa Antonio, Guerci Antonio (1982). Various uses of the castor oil plant (Ricinus communis L.) a review. Journal of Ethnopharmacology.

[28] Jena J, Gupta AK. *Ricinus communis* Linn: a phytopharmacological review. Int J Pharm Pharm Sci. 2012;4:25–9.

[29] Maroyi A (2011). An ethnobotanical survey of medicinal plants used by the people in Nhema communal area, Zimbabwe. J Ethnopharmacol.

[30] Mabona U, Van Vuuren SF (2013). Southern African medicinal plants used to treat skin diseases. S Afr J Bot.

[31] Bhat RB. Medicinal plants and traditional practices of Xhosa people in the Transkei region of Eastern Cape, South Africa. Indian J Tradit Knowl. 2014;13:292–8.

[32] Morekhure-Mphahlele RWWF (2015). Wiebke Grote: characterisation of vumba and ubumba clays used for cosmetic purposes. S Afr J Sci.

[33] Molefe O: Physico-chemical characterization of African traditional cosmetics produced by the Ovahimba tribes of northern Namibia. 2015.

[34] Volpato Gabriele, Kourková Pavlína, Zelený Václav (2012). Healing war wounds and perfuming exile: the use of vegetal, animal, and mineral products for perfumes, cosmetics, and skin healing among Sahrawi refugees of Western Sahara. Journal of Ethnobiology and Ethnomedicine.

[35] Zhang S, Lo ECM, Chu CH. Traditional oral health beliefs and practices of Bulang people in Yunnan, China. J Investig Clin Dent. 2018;9.e12281. 10.1111/jicd.12281. 28685949

[36] Fred-Jaiyesimi Adediwura, Ajibesin K. K., Tolulope Odeyemi, Gbemisola Ogundokun (2014). Ethnobotanical studies of folklore phytocosmetics of South West Nigeria. Pharmaceutical Biology.

[37] Afolayan Anthony J., Grierson Donald S., Mbeng Wilfred O. (2014). Ethnobotanical survey of medicinal plants used in the management of skin disorders among the Xhosa communities of the Amathole District, Eastern Cape, South Africa. Journal of Ethnopharmacology.

[38] Kuete Victor, Viertel Katrin, Efferth Thomas (2013). Antiproliferative Potential of African Medicinal Plants. Medicinal Plant Research in Africa.

[39] Mathabe MC, Nikolova RV, Lall N, Nyazema NZ (2006). Antibacterial activities of medicinal plants used for the treatment of diarrhoea in Limpopo Province, South Africa. J Ethnopharmacol.

[40] Güzel Y, Güzelşemme M, Miski M (2015). Ethnobotany of medicinal plants used in Antakya: a multicultural district in Hatay Province of Turkey. J Ethnopharmacol.

[41] Omwenga EO, Hensel A, Shitandi A, Goycoolea FM (2015). Ethnobotanical survey of traditionally used medicinal plants for infections of skin, gastrointestinal tract, urinary tract and the oral cavity in Borabu sub-county, Nyamira county, Kenya. J Ethnopharmacol.

[42] Agyare C, Asase A, Lechtenberg M, Niehues M, Deters A, Hensel A (2009). An ethnopharmacological survey and in vitro confirmation of ethnopharmacological use of medicinal plants used for wound healing in Bosomtwi-Atwima-Kwanwoma area, Ghana. J Ethnopharmacol.

[43] Bhowmik D: *Aloe Vera*: The Miracle Plant Its Medicinal and Traditional Uses in India, vol. 1; 2012.

[44] Rodrigues Luiza lucy Oliveira, de Oliveira Ana Carolina Leal, Tabrez Shams, Shakil Shazi, Khan Mohammad Imran, Asghar Muhammad Nadeem, Matias Bianca Dias, Batista Joysa Michelle Alves da Silva, Rosal Marinilva Modesto, de Lima Marylia Maria Duarte Fulgencio, Gomes Sávia Ribeiro Ferreira, de Carvalho Rodrigo Mendes, de Moraes Germano Pinho, de Alencar Marcus Vinicius Oliveira Barros, Islam Muhammad Torequl, Melo-Cavalcante Ana Amélia de Carvalho (2018). Mutagenic, antioxidant and wound healing properties of Aloe vera. Journal of Ethnopharmacology.

[45] Amoo SO, Aremu AO, Van Staden J (2014). Unraveling the medicinal potential of south African *Aloe* species. J Ethnopharmacol.

[46] Woldemedhin B, Nedi T, Shibeshi W, Sisay M. Evaluation of the diuretic activity of the aqueous and 80% methanol extracts of the root of *Euclea divinorum* Hiern (Ebenaceae) in Sprague Dawley rats. J Ethnopharmacol. 2017;202:114–21. 10.1016/j.jep.2017.01.015 28089738

[47] Otang WM, Afolayan AJ (2016). Antimicrobial and antioxidant efficacy of *Citrus limon* L. peel extracts used for skin diseases by Xhosa tribe of Amathole District, Eastern Cape, South Africa. S Afr J Bot.

[48] Otang WM, Grierson DS, Afolayan AJ. A survey of plants responsible for causing allergic contact dermatitis in the Amathole District, Eastern Cape, South Africa. S Afr J Bot. 2015;97:32–9. 10.1016/j.jep.2014.10.002 25311272

[49] Rankoana SA, Nel K, Mothibi K, Mothiba TM, Mamogobo P, Setwaba M (2015). The use of indigenous knowledge in primary health care: a case study of Makanye community in Limpopo Province, South Africa. Afr J Phys, Health Educ, Recreation Dance.

[50] Shenefelt PD, Shenefelt DA (2014). Spiritual and religious aspects of skin and skin disorders. Psychol Res Behav Manag.

[51] White P (2015). The concept of diseases and health care in African traditional religion in Ghana. HTS Theological Stud.

[52] Workneh T, Emirie G, Kaba M, Mekonnen Y, Kloos H. Perceptions of health and illness among the Konso people of southwestern Ethiopia: persistence and change. J Ethnobiol Ethnomed. 2018;14:18. 10.1186/s13002-018-0214-y. 10.1186/s13002-018-0214-y PMC6389056 29482630

[53] Quave CL, Pieroni A, Bennett BC. Dermatological remedies in the traditional pharmacopoeia of Vulture-Alto Bradano, inland southern Italy. J Ethnobiol Ethnomed. 2008;4. 10.1186/1746-4269-1184-1185. 10.1186/1746-4269-4-5 PMC2275234 18254949

[54] Reyes-García V. The relevance of traditional knowledge systems for ethnopharmacological research: theoretical and methodological contributions. J Ethnobiol Ethnomed. 2010;6. 10.1186/1746-4269-1186-1132. 10.1186/1746-4269-6-32 PMC2993655 21083913

[55] Gebashe F., Moyo M., Aremu A.O., Finnie J.F., Van Staden J. (2019). Ethnobotanical survey and antibacterial screening of medicinal grasses in KwaZulu-Natal Province, South Africa. South African Journal of Botany.

[56] Eller JD. Ethnicity, Culture, and 'The Past'. Michigan Quarterly Rev. 1997;36(4). http://hdl.handle.net/2027/spo.act2080.0036.411.

[57] Yeşilada Erdem (2002). Biodiversity in Turkish Folk Medicine. Biodiversity.

